# A high-throughput microfluidic bilayer co-culture platform to study endothelial-pericyte interactions

**DOI:** 10.1038/s41598-021-90833-z

**Published:** 2021-06-09

**Authors:** Miles T. Rogers, Ashley L. Gard, Robert Gaibler, Thomas J. Mulhern, Rivka Strelnikov, Hesham Azizgolshani, Brian P. Cain, Brett C. Isenberg, Nerses J. Haroutunian, Nicole E. Raustad, Philip M. Keegan, Matthew P. Lech, Lindsay Tomlinson, Jeffrey T. Borenstein, Joseph L. Charest, Corin Williams

**Affiliations:** 1 The Charles Stark Draper Laboratory Inc., 555 Technology Square, Cambridge, MA 02139 USA; 2 Pfizer, Inc., 1 Portland St, Cambridge, MA 02139 USA; 3 Present Address: Raytheon BBN Technologies, Synthetic Biology, 10 Moulton St, Cambridge, MA 02138 USA; 4 Present Address: Microsoft Corporation, 1 Memorial Drive, Cambridge, MA 02142 USA; 5 Present Address: Department of Biology, Northeastern University, 360 Huntington Ave, Boston, MA 02115 USA; 6 Present Address: Department of Biomedical Engineering, University of Wisconsin Madison, 1550 Engineering Dr, Madison, WI 53706 USA

**Keywords:** Biological techniques, Biotechnology, Cell biology, Engineering

## Abstract

Microphysiological organ-on-chip models offer the potential to improve the prediction of drug safety and efficacy through recapitulation of human physiological responses. The importance of including multiple cell types within tissue models has been well documented. However, the study of cell interactions in vitro can be limited by complexity of the tissue model and throughput of current culture systems. Here, we describe the development of a co-culture microvascular model and relevant assays in a high-throughput thermoplastic organ-on-chip platform, PREDICT96. The system consists of 96 arrayed bilayer microfluidic devices containing retinal microvascular endothelial cells and pericytes cultured on opposing sides of a microporous membrane. Compatibility of the PREDICT96 platform with a variety of quantifiable and scalable assays, including macromolecular permeability, image-based screening, Luminex, and qPCR, is demonstrated. In addition, the bilayer design of the devices allows for channel- or cell type-specific readouts, such as cytokine profiles and gene expression. The microvascular model was responsive to perturbations including barrier disruption, inflammatory stimulation, and fluid shear stress, and our results corroborated the improved robustness of co-culture over endothelial mono-cultures. We anticipate the PREDICT96 platform and adapted assays will be suitable for other complex tissues, including applications to disease models and drug discovery.

## Introduction

The current drug development process is limited by the imprecise translation of assay results in early discovery phases to the prediction of human response. This is partly due to the limited availability of human-based models that replicate human disease and tissue-specific environments. Plate-based assays do not mimic the complexity of human physiology which requires multiple cell types, fluid flow, and appropriate cell-to-volume ratios. Although preclinical animal models recapitulate the complexity of the human, the translation from animal to human is not always predictive. Each of these obstacles results in unexpected clinical trial failures with significant financial and opportunity losses. It is estimated that a single new molecular entity or first in class drug can require a decade of development and $2.5B to bring to market^1^.

Current efforts are working to improve translation from early discovery and preclinical models to better predict clinical trial success. Examples of these models include human tissue spheroids and immunodeficient mice^2,3^. In addition, a research area that offers much promise is human-based microphysiological systems (MPS), otherwise known as organ-on-chip systems. This field of research has seen a steady increase in public and private funding in the last decade^4^. Progress in the field to date has produced MPS devices that allow for control over the complex biology found in human tissues and organs, such as incorporation of fluid flow to provide media or nutrient replenishment, biomechanical cues such as fluid shear stress (FSS) or stretch to mimic native tissue environments^5,6,7,8,9^, and recapitulation of cell-to-fluid ratios in the body to allow for translational readouts^10^. High replicate systems, such as the platform described in this work, can facilitate compound screening and high-throughput readouts.

There is a significant need to develop human vascular models for toxicity screening to prevent drug induced vascular injury^7,9^ as well as for the testing of novel therapies for a variety of vascular diseases, such as atherosclerosis, hypertension, diabetic retinopathy, vascular fibrosis, Hutchinson-Gilford Progeria Syndrome, or vascular malformation disorders^8^. Important variables to consider in the design of vascular models include cell types and micro-environmental cues. Endothelial cells (ECs) from different organs have unique phenotypes^11,12^ and associate with specific mural cell populations (smooth muscle cells, pericytes, fibroblasts), depending on vessel caliber and location^13^. It is well documented that the interaction of ECs with mural cells significantly influences their behavior. For example, in 3D gel systems, the inclusion of mesenchymal stromal cells, fibroblasts, or pericytes (PCs) is critical for angiogenesis, lumen formation, and long-term vessel stability^14,15^. Co-culture of ECs with smooth muscle cells increases LDL uptake by ECs^16^, alters gene expression^17^, and inhibits TNFα-mediated EC activation^18^. In blood–brain barrier models, the inclusion of PCs is critical for enhancing barrier function of the microvascular endothelium^19^. The development of co-culture vascular MPS-based models in which human EC populations can be partnered with appropriate mural cells would help to bridge the current gaps in translation of animal model responses to the clinic for vascular targets and safety de-risking.

In this paper, we introduce a model of the human microvasculature using human retinal microvascular ECs and PCs in a high-throughput MPS platform. The platform, PREDICT96, is first-in-class for its combination of 96 arrayed bilayer membrane-based devices, high content imaging capabilities, physiologically relevant pumping, and thermoplastic materials^20^. Recently, the utility of the PREDICT96 platform was demonstrated for a human liver model^21,22^. Here, we show compatibility of the PREDICT96 platform with a variety of standard assays for vascular models, including: the macromolecular tracer assay to assess tissue permeability and barrier function, image-based screening of endothelial monolayers for tissue models cultured under various treatments, multiplexed cytokine profiling by Luminex, and gene expression by quantitative polymerase chain reaction (qPCR). Given the bilayer design of the PREDICT96 devices, a unique advantage of the platform is the ability to extract cell type- or channel-specific readouts, such as that demonstrated by Luminex and qPCR results. We expect the PREDICT96 platform to be advantageous for studying the interactions between multiple cell types in various complex tissue models beyond the microvasculature system described here.

## Results

### The microvascular co-culture model in the PREDICT96 platform

**Figure 1:**
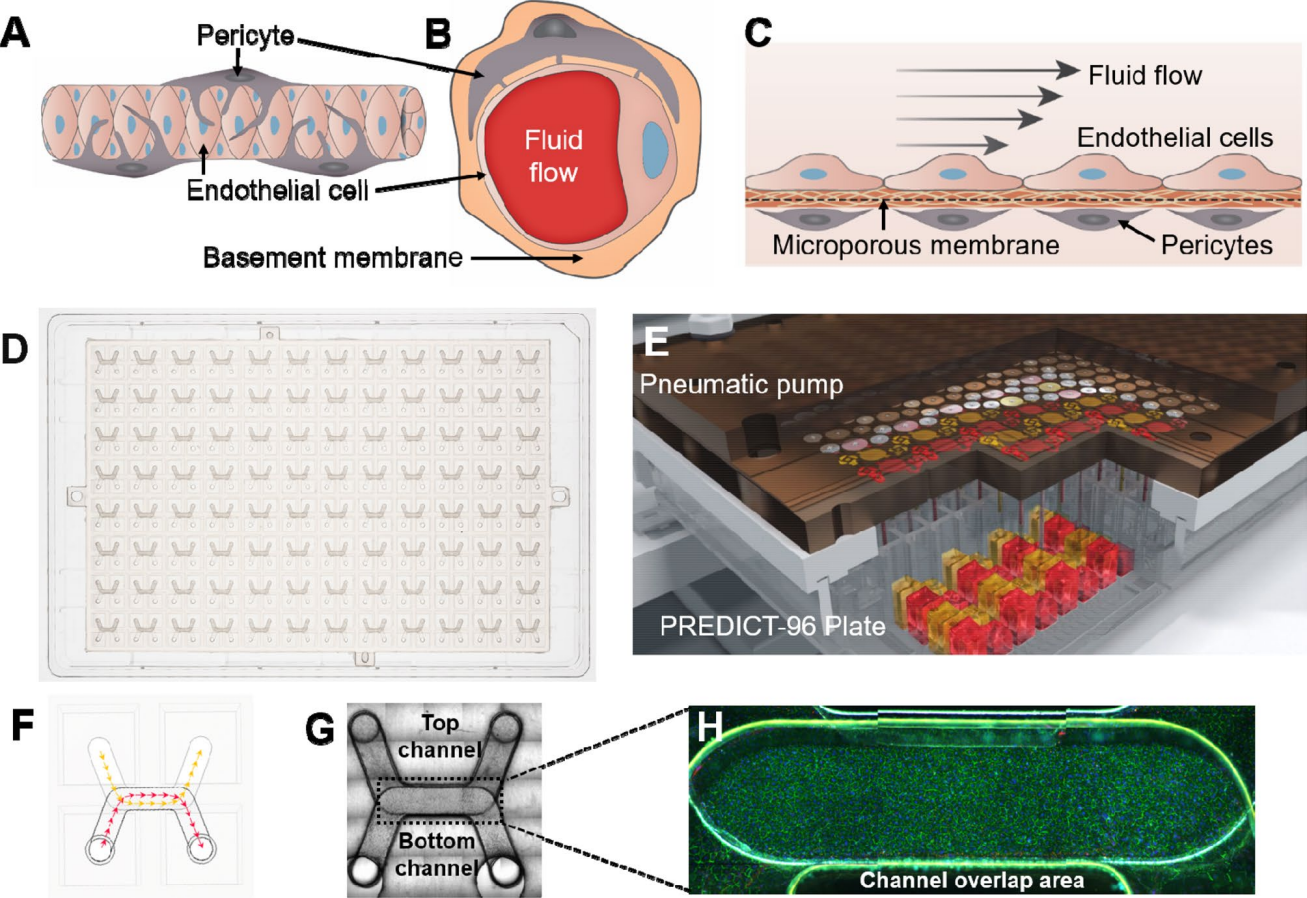
Development of the microfluidic microvascular co-culture model in the PREDICT96 platform. (**A**) Side-view schematic of the microvasculature. Capillaries are surrounded by pericytes which, under healthy conditions, help stabilize and mature the endothelium. (**B**) Cross-section schematic showing interaction of endothelial cells (ECs) and pericytes (PCs) through basement membrane. (**C**) Side-view cross-section schematic of the vascular model in the bilayer microfluidic device. ECs and PCs are cultured on either side of a microporous membrane coated with extracellular matrix, which allows interaction between the two cell types. (**D**) Top-view of the PREDICT96 plate, containing an array of 96 bilayer microfluidic devices that interfaces with a 384 well plate top. (**E**) Schematic of PREDICT96 custom pneumatic pump lid, containing 192 individual pumps that control fluid flow in each channel of the 96 bilayer devices. (**F**) A single PREDICT96 device corresponds to 4 wells of the 384 well plate with architecture allowing for culture and fluid flow in separate top and bottom channels, which overlap in the device center. (**G**) Top-view bright field image of a single device, with channel overlap area indicated as the region of interest (dotted rectangle). (**H**) Representative image of ECs stained for PECAM-1 (green) and Hoechst (blue) in channel overlap area.

A reproducible model of the human retinal microvasculature, comprised of ECs and PCs, was created in the PREDICT96 platform. In the capillary system, ECs and PCs are in close proximity, and directly contact each other through a thin layer of basement membrane^13,23^ (Fig. 1A,B). Our microvascular model allowed for interaction between the cell types via culturing the ECs and PCs on opposing sides of a 10 μm-thick microporous membrane within the bilayer microfluidic device (Fig. 1C). Furthermore, separation of ECs and PCs by a physical barrier permitted channel- or cell population-specific stimuli and readouts. The bilayer microfluidic devices are arrayed in the PREDICT96 plate (Fig. 1D), allowing for the culture of up to 96 individual tissue models on a single plate. The PREDICT96 pump “lid”, containing 192 individual pumps (1 pump for each channel of the 96 devices), was used to provide fluid flow (Fig. 1E) for nutrient exchange, mixing (as in the permeability assay), or application of FSS across the EC monolayer. The PREDICT96 plate utilizes a bottomless 384 well interface, where each inlet and outlet port of the top and bottom channels is accessible via one well, for a total of 4 wells per device (Fig. 1F). Each channel has a U-shape design (top channel membrane surface area 8.5 mm^2^, bottom channel membrane surface area 6.9 mm^2^) with an overlap area in the middle (3.7 mm^2^), which is the region of interest for imaging (Fig. 1G,H). It is also within this channel overlap area that ECs and PCs interacted across the membrane.

### Co-cultures maintain long-term viability and cell coverage in PREDICT96 plates

**Figure 2:**
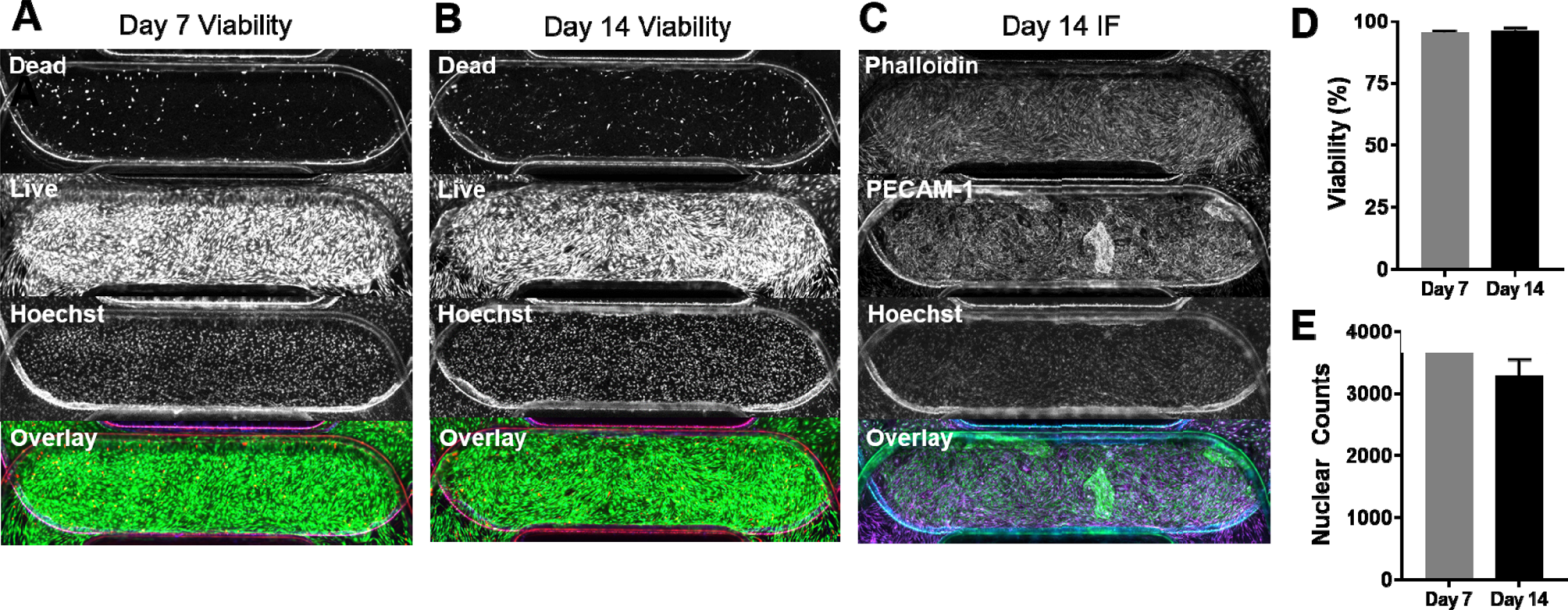
Long-term co-culture viability is high in PREDICT96 devices. Representative co-culture devices assessed for viability at (**A**) day 7 and (**B**) day 14 with split channels in descending order: red, green, blue, overlay. (**C**) Representative co-culture device stained for actin fibers (Phalloidin) and ECs (PECAM-1) on day 14 shows good coverage for both cell types. (**D**) Viability was greater than 95% at days 7 and 14. (**E**) Total cell numbers as determined by nuclear counts in channel overlap at day 7 and 14 did not differ significantly. N = 2 at day 7; N = 4 at day 14.

To assess long-term viability in PREDICT96 microfluidic devices, EC-PC co-cultures were maintained for 2 weeks. Devices were assessed by live-dead staining at 7 and 14 days post-PC seeding (Fig. 2A,B). Devices fixed for immunofluorescence at day 14 positively stained for actin fibers (Phalloidin) and the EC marker PECAM-1, highlighting full coverage for both cell types in the channel overlap (Fig. 2C). Enhanced PECAM-1 staining was occasionally observed in areas with increased cell density, a typical observation for this EC source in our experience. Co-culture viability was greater than 95% at both time points (95.5 ± 0.6% and 96.1 ± 1.2%, respectively) (Fig. 2D). There was also no significant difference in total cell numbers in the channel overlap area when comparing day 7 to day 14 (3,670 ± 155 cells and 3292 ± 264 cells, respectively) (Fig. 2E).

### Compatibility of PREDICT96 with macromolecular permeability assays and detection of barrier disruption

**Figure 3:**
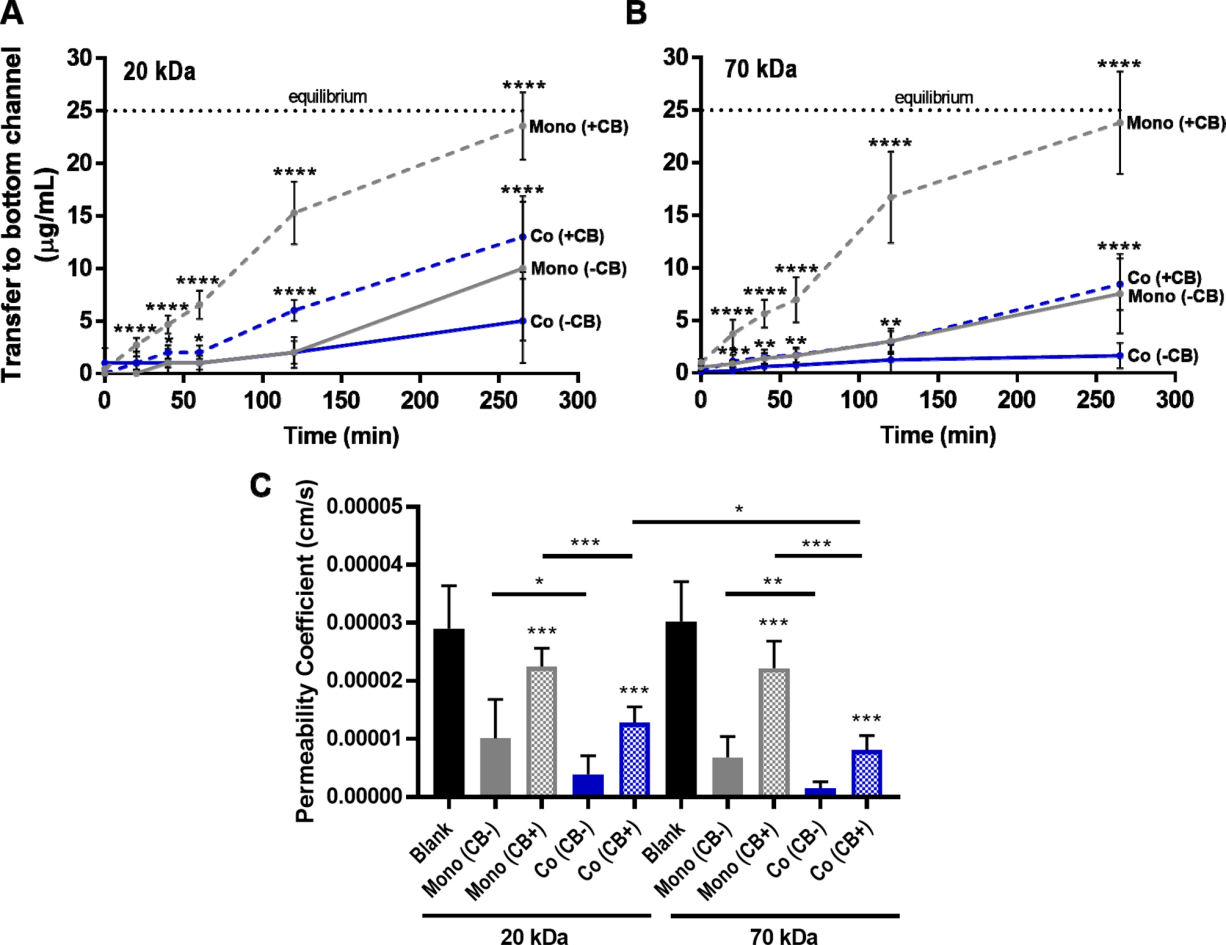
Permeability assays in PREDICT96 vascular models. FITC-dextran was loaded at a concentration of 50 μg/ml into the top channel of devices and transfer to the bottom channel was tracked over time. Note that equilibrium between both channels would be at 25 μg/mL (indicated by black dotted line). Both EC mono-culture (Mono) and co-culture with PC (Co) were assessed. Treatment with cytochalasin B (CB) was used to disrupt the barrier (added at t = 0). Transfer of (**A**) 20 kDa FITC-dextran and (**B**) 70 kDa FITC-dextran across the vascular barrier was measured at various intervals over 260 min. (**C**) Permeability coefficients were calculated at the 260 min time point for each condition assayed in the PREDICT96 plate. N = 8–10 technical replicates per condition. Asterisks in A and B indicate significant difference in + CB treated conditions compared to -CB controls at corresponding time points.

A gold standard for assessing endothelial barrier function is the macromolecular permeability assay^24^. After establishing EC mono-cultures and EC-PC co-cultures in PREDICT96 devices, media containing 20 kDa or 70 kDa FITC-dextran tracer molecules was added to the top channel of the devices, and transfer to the bottom channel was measured over the course of 260 min (Fig. 3A,B). For permeability coefficients calculated at the 260 min time point, there was no significant difference between 20 kDa FITC-dextran and 70 kDa FITC-dextran in the mono- and co-cultures, with the exception of cytochalasin B-treated co-cultures (Fig. 3C). For both dextrans, co-cultures showed significantly increased barrier function over mono-cultures, and treatment with the mycotoxin cytochalasin B significantly decreased barrier function, as indicated by permeability coefficients calculated at the 260 min time point. For non-treated samples, the permeability coefficient for 20 kDa FITC-dextran was 3.9 × 10^–6^ cm/s in the co-culture and 1.0 × 10^–5^ cm/s in the mono-culture, while the 70 kDa FITC-dextran transferred at a rate of 1.5 × 10^–6^ cm/s in the co-culture vs. 6.8 × 10^–6^ cm/s in the mono-culture (Fig. 3C). For cytochalasin B-treated samples, 20 kDa permeability coefficients significantly increased to 1.3 × 10^–5^ cm/s in the co-culture and 2.3 × 10^–5^ in the mono-culture, and 70 kDa increased to 8.1 × 10^–6^ cm/s in the co-culture and 2.2 × 10^–5^ cm/s in the mono-culture. Significant differences in transfer were detectable as early as 20 min after disrupting the mono-culture with cytochalasin B for both dextrans, while the effect of disrupting the co-culture was significant at 20 min for 70 kDa dextran, and 60 min for 20 kDa dextran (Fig. 3A,B). Of note, permeability coefficients among the technical replicates for each condition did not deviate more than one order of magnitude (heat map shown in Supplementary Fig. S1).

### PREDICT96 facilitates image-based screening of EC monolayers with various co-culture media formulations

In order to create a co-culture vascular model in which different perturbations such as inflammation or disease can be assessed, a low serum media formulation is often required. However, maintaining EC monolayers in low serum or other starvation media conditions is challenging. We screened 10 different media formulations for the co-culture model to determine which conditions would adequately support EC monolayers. We included two microvascular EC populations (dermal and retinal) in mono-culture and in co-culture with PCs. This allowed for two technical replicates per condition on a single PREDICT96 plate. Prior to seeding the PCs into the co-culture devices and subsequent media screening, we ran a permeability assay and found no significant difference in barrier function between the two EC sources (Supplementary Fig. S2).

**Figure 4:**
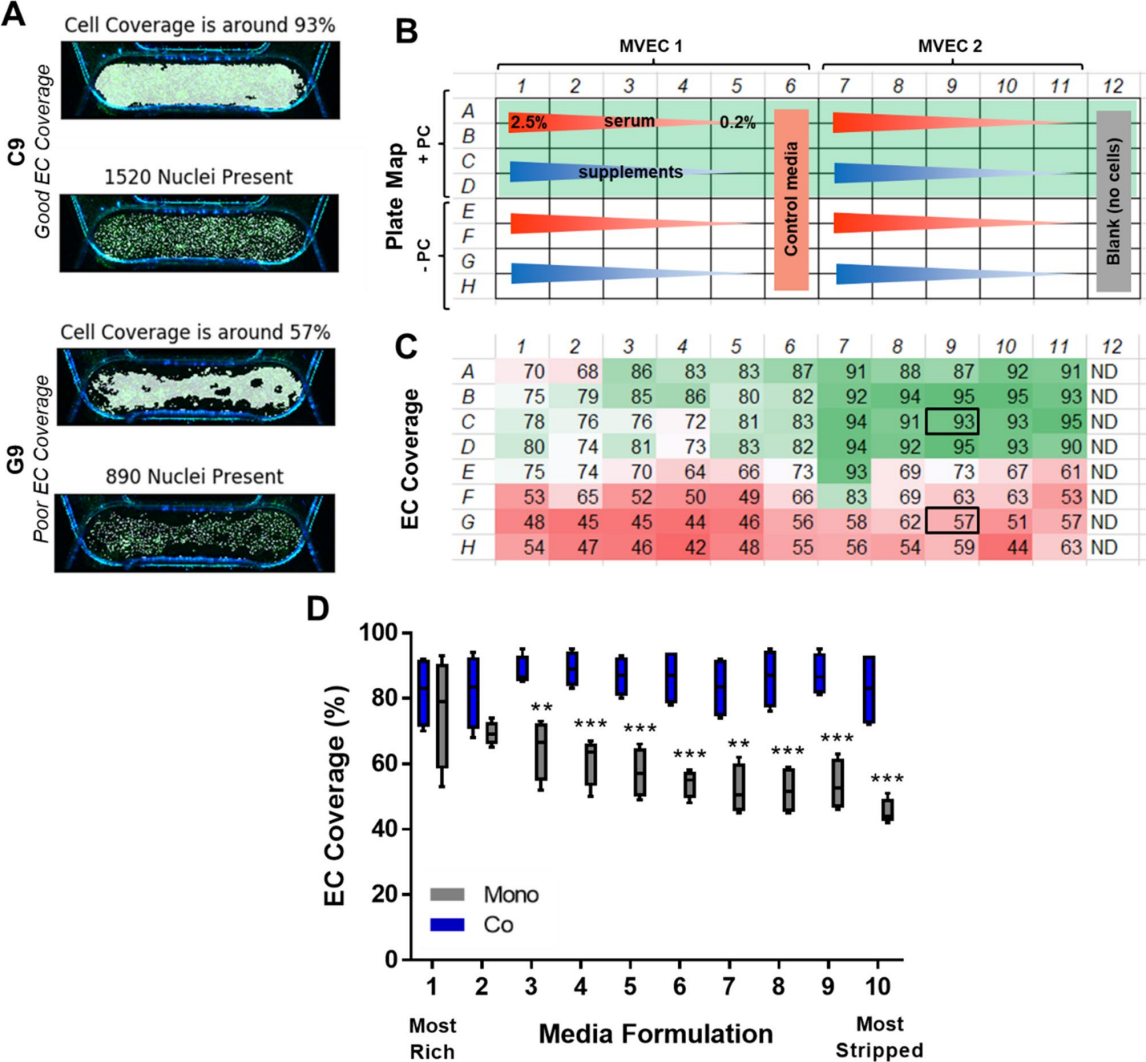
Quantification of EC monolayer coverage in co-culture media screening. (**A**) Representative outputs from custom code for conditions with “good” endothelial cell coverage and “poor” coverage. (**B**) Plate map for conditions assessed: 10 media formulations with decreasing serum concentrations (red gradient) or 0.2% serum and decreasing supplements (blue gradient). The top half of the plate assessed co-culture (rows A–D) with corresponding mono-culture conditions in the bottom half of the plate (rows E–H). (**C**) Automated coverage and nuclear count measurements for channel overlap area in devices across the PREDICT96 plate. Note improved coverage in co-culture (rows **A**–**D**) vs. mono-culture (rows E–H) conditions. Note that column 12 was left blank for permeability assay and therefore no cells were detected (ND). (**D**) EC coverage as a function of media formulation (N = 4 per condition). The ten unique medias are indicated as 1 being the most rich and 10 as the most stripped down. Co-culture with PC maintained EC monolayers even in severe starvation formulations.

At the end of the experiment, images were taken of the channel overlap area across the entire PREDICT96 plate (with the exception of column 12, which was left blank for permeability assay controls), and used to assess EC monolayers for each condition via staining for PECAM-1 (Supplementary Fig. S3). Our custom code processed the images and provided outputs of EC coverage as well as total nuclear counts in the channel overlap area. Representative outputs for “good” and “poor” EC coverage are shown in Fig. 4A. The plate map for the various conditions and resulting measurements of EC coverage are shown in Fig. 4B,C, respectively. Generally, we found that the dermal MVEC lot used in this experiment formed more robust monolayers compared to retinal MVEC, and that EC monolayers were significantly improved in co-culture vs. mono-culture conditions (Fig. 4C,D). In fact, co-culture with PCs stabilized the EC monolayers independent of the media formulations that were explored (Fig. 4D).

For the subsequent experiments described in this paper, we continued to use retinal microvascular ECs as a tissue-matched source to the retinal PCs, and chose media formulation 3 as the starvation media.

### Channel-specific readouts from PREDICT96 devices demonstrated by Luminex and qPCR

**Figure 5:**
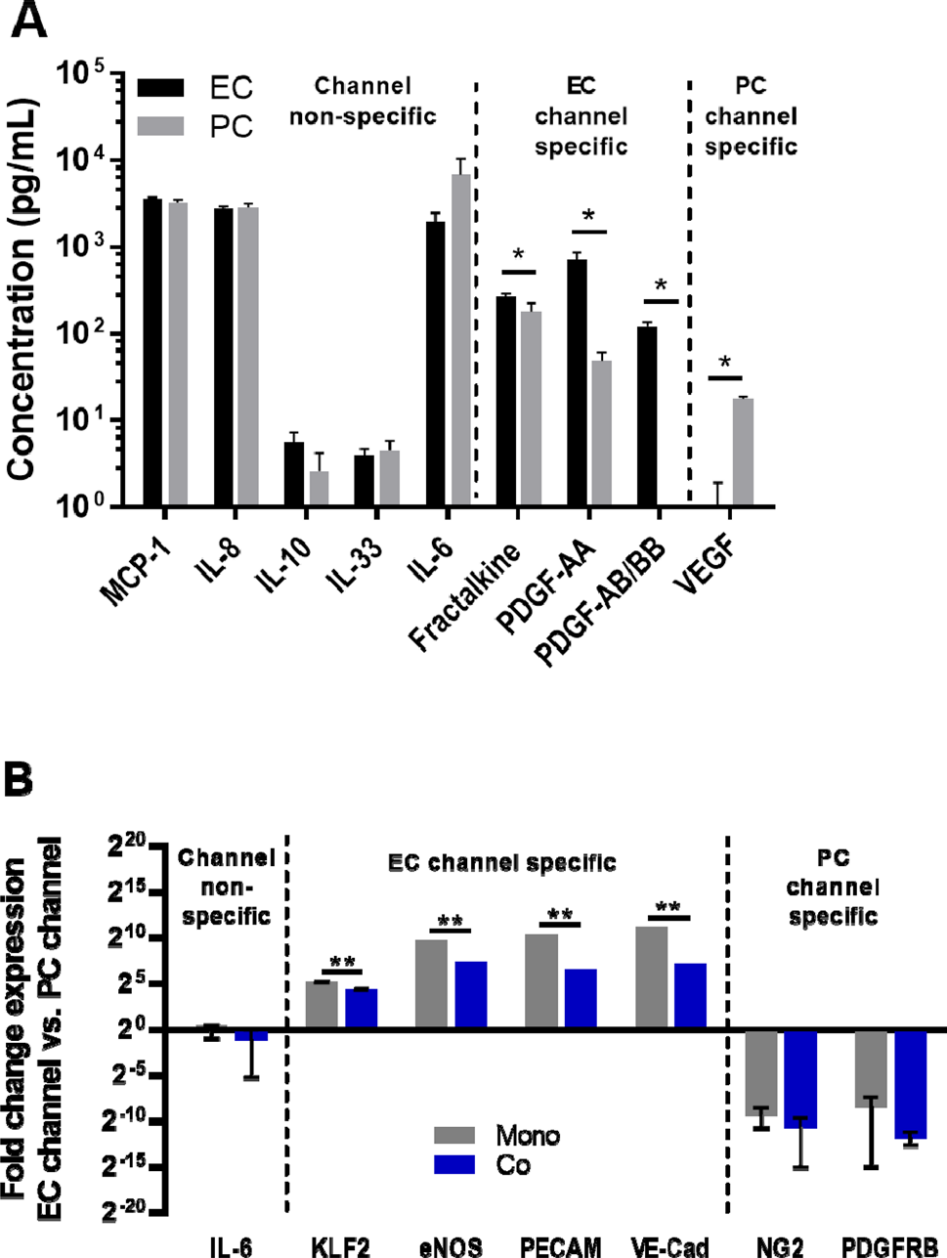
Detection of channel- and cell-type specific responses in PREDICT96 co-cultures. (**A**) Select data shown from 16-plex Luminex kit run on media collected from EC and PC channels after 24 h. Note that some secreted factors are expressed in both channels while others are differentially expressed in EC or PC channels. (**B**) Gene expression for cells isolated from EC and PC channels of devices show that EC markers are significantly enriched in EC channels, while PC markers are significantly enriched in PC channels. Note that IL-6 is expressed by both cell types (secreted and gene). Enrichment of EC markers in the EC channel was significantly decreased in co-culture compared to mono-culture, indicated potential inter-channel crosstalk.

In order to monitor the responses of different cell types in a co-culture model, it is desirable to isolate components from each culture channel for analysis. To this end, two different assays were used to evaluate the potential for independently analysis of channels/cell populations in the PREDICT96 devices: soluble factors in the media and gene expression. A Luminex assay was used to examine secreted soluble factors present in the media in the EC and PC channels after 24 h. Several analytes that were not specific to either cell type included MCP-1, IL-8, IL-10, IL-33, and IL-6 (Fig. 5A). However, we found that fractalkine/CX3CL1, PDGF-AA and PDGF-AB/BB were significantly higher in the EC channel whereas VEGF was significantly higher in the PC channel.

In order to determine whether transcriptional information could be independently analyzed from each channel, ECs and PCs were harvested from the top and bottom channels respectively, followed by RNA isolation and qPCR. IL-6 gene expression was not channel-specific, similar to our Luminex result. EC-specific genes for KLF2, eNOS, PECAM-1, and VE-Cadherin were significantly elevated in the EC channel compared to the PC channel, while PC-specific genes for NG2 and PDGFRβ were preferentially expressed in the PC channel (Fig. 5B). Notably, the increase in channel-specific expression was approximately tenfold greater in the mono-culture than in the co-culture, indicating that there may be some cross-talk between channels in the co-cultures.

### Inflammatory assay shows improved stability of the endothelium in co-culture and channel-specific cytokine profiles

**Figure 6:**
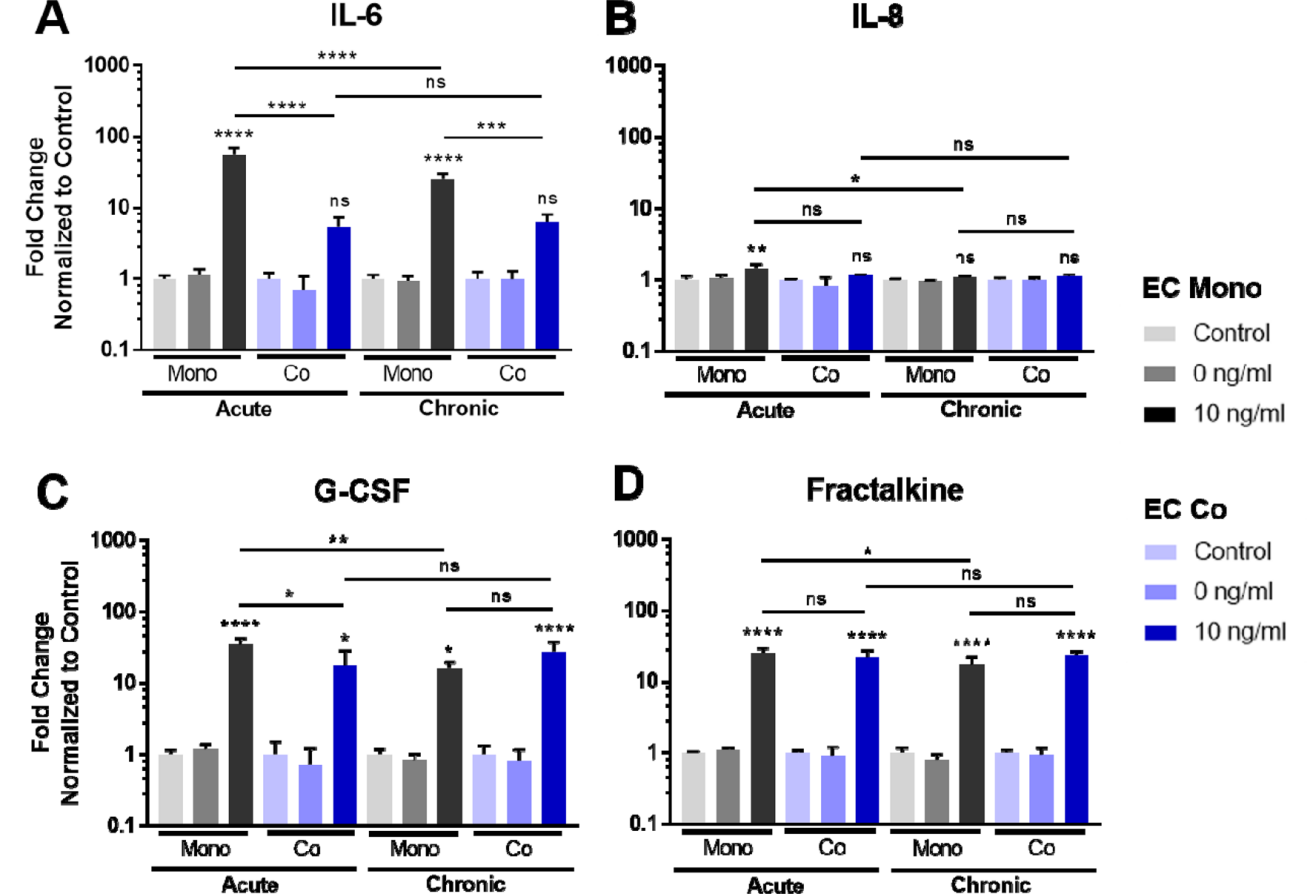
Co-culture model response to inflammatory insult—cytokine profiles from EC channels. Select secreted cytokines in media isolated from the EC channel were analyzed by Luminex, with a comparison of EC mono- vs. EC co-cultures in acute and chronic TNFα stimulation conditions. N = 3 per condition.

To determine the response of the co-culture vascular model to disease or inflammatory perturbations, we performed an inflammation assay using TNFα applied to device channels containing ECs. We studied both “acute” (10 ng/ml TNFα stimulation for 4 h followed by 20 h recovery) and “chronic” dosing (24 h TNFα stimulation), with media collected from each channel at the 24 h time point immediately prior to fixing and staining the samples. Luminex analysis revealed that several cytokines were increased after TNFα stimulation: in the acute treatment, IL-6, IL-8, G-CSF, and Fractalkine were all significantly increased compared to controls in the EC channel in the mono-cultures, as well as in the co-cultures for G-CSF and Fractalkine (Fig. 6). In the case of ECs from the co-culture, IL-6 was not significantly increased compared to control samples, and was significantly lower compared to corresponding TNFα-treated EC mono-culture conditions (Fig. 6A). In addition, G-CSF was significantly lower in the co-culture EC channel compared to the EC mono-culture in the acute condition (Fig. 6C). These results show that differences in acute vs. chronic treatment as well as mono- vs. co-cultures can be measured in our system.

Representative images of the ECs post-perturbation are shown in Supplementary Fig. S4A. EC monolayer disruption was evident with 10 ng/ml TNFα, particularly for the chronic stimulation. EC coverage in the chronic stimulation condition decreased from 93 to 60% and 96% to 74% in mono- and co-cultures, respectively (Supplementary Fig. S4B). However, EC coverage was significantly improved in the co-culture vs. mono-culture TNFα-treated conditions. In the acute inflammation study, there was still a significant loss of EC coverage in the mono-culture after TNFα treatment. However, co-cultures had similar coverage compared to controls (Supplementary Fig. S4B). Taken together with the Luminex results, these findings indicate that the presence of PCs helped stabilize ECs during acute inflammatory insult.

### Channel-specific gene expression in response to fluid shear stress

**Figure 7:**
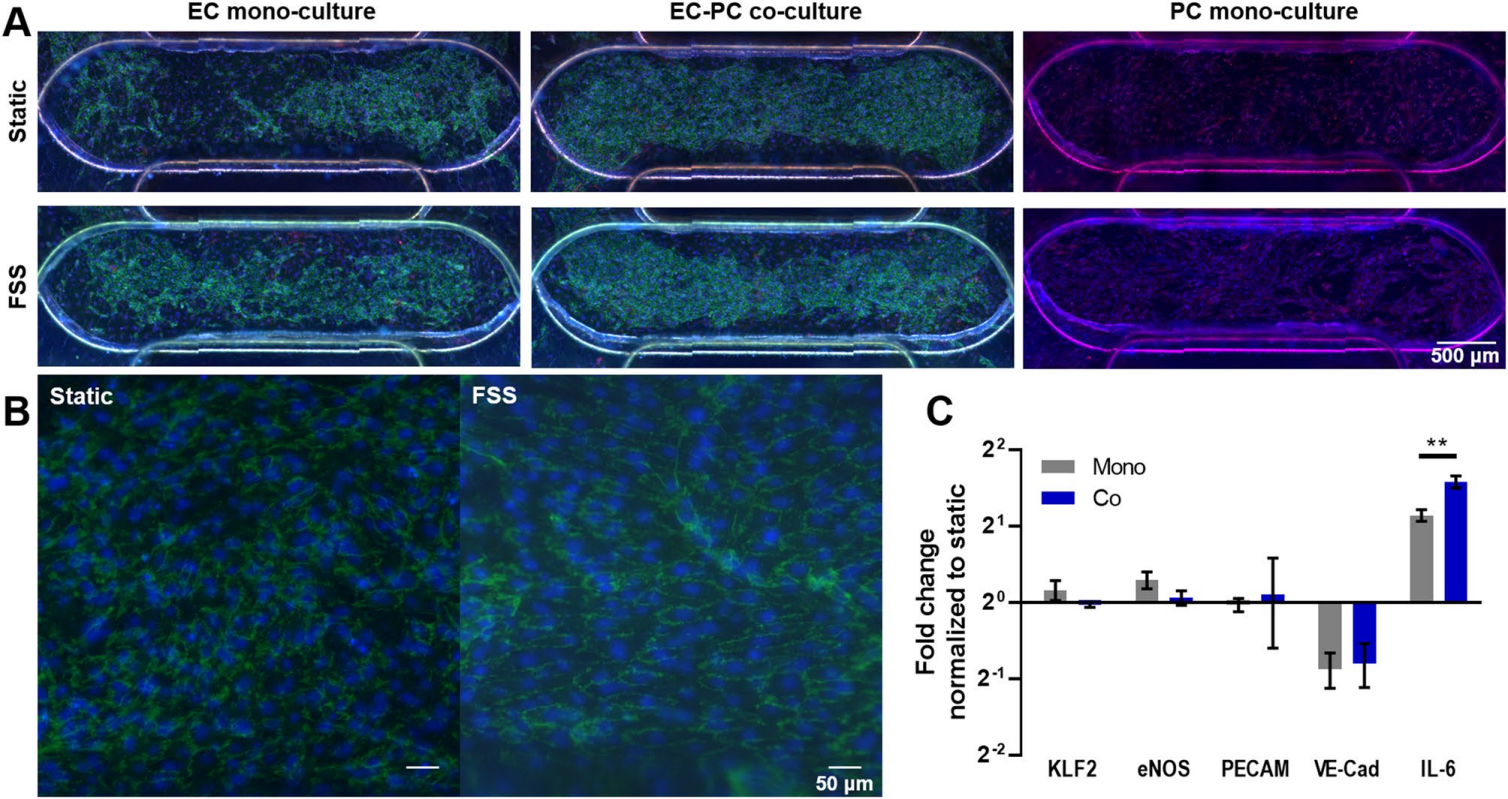
Vascular model response to low fluid shear stress (FSS). (**A**) Representative images of devices 24 h after exposure to low FSS. (**B**) Higher magnification shows some alignment of EC in response to fluid flow. (**C**) Gene expression of ECs from mono- and co-cultures showed that IL-6 was significantly increased in flow, while VE-CAD expression was significantly decreased. IL-6 was also significantly increased in co-culture compared to mono-culture under flow.

As FSS is an important regulator of EC response^25^, we applied low shear at 0.5 dyn/cm^2^ to the top channel of PREDICT96 devices for 24 h in EC mono-culture, EC-PC co-culture, and PC mono-culture conditions. Similar to previous observations, the presence of PC improved EC monolayers, and this was independent of applied FSS (Fig. 7A). Qualitatively, some alignment of the ECs with the direction of FSS were observed in the channel, while static ECs remained disorganized (Fig. 7B).

In addition to phenotypic characterization using immunofluorescence, analysis of transcriptional changes was used to determine the impact of co-culture on gene expression. ECs subjected to low FSS had a significant decrease in VE-Cadherin and a significant increase in pro-inflammatory cytokine IL-6 gene expression (Fig. 7C). Both of these responses have been documented by other groups as typical of ECs subjected to FSS as low as 3.5 dynes/cm^2^^26,27^. ECs grown in co-culture had a small but significant increase in IL-6 expression compared to mono-culture (Fig. 7C). Overall, these results demonstrate the compatibility of the PREDICT96 platform with genetic assays and the ability to investigate cell type specific gene expression patterns in co-culture models.

## Discussion

The work presented in this paper summarizes the development of a robust microvascular co-culture model in a high-throughput bilayer microfluidic platform. In the process of developing and characterizing the model, a variety of standard biological assays were adapted to the PREDICT96 system. These capabilities include the macromolecular permeability assay to assess barrier tissue function, image-based screening assays of cell markers or phenotypes, cytokine profiling by Luminex, and gene expression by qPCR. These assays are amenable to the high throughput nature of the PREDICT96 platform and compatible with the low cell numbers and media volumes extracted from devices. Furthermore, by separating the ECs and PCs by a microporous membrane that permitted cell–cell communication but limited cell translocation, it was possible to carry out channel-specific assays, such as the assessment of the EC monolayer by immunofluorescent staining, secreted factor profiling in each channel, and gene expression in cell populations isolated from each channel. The ability to study cell type-specific responses using a variety of assays provides a powerful toolkit for future developments of the vascular model or other multicellular tissue models.

Often, microvascular models are generated in 3D gel systems^15^; however, the assays that can be performed in 3D, particularly with mixed cell populations, are cumbersome and limited in their throughput and ease of imaging. Permeability can also be challenging to quantify in 3D models. The ability to “flatten” the capillary model into 2D simplifies the assessment of EC monolayer barrier function and imaging-based assays. (Fig. 1A–C). To this end, Transwell culture inserts are a common co-culture tool^28^, but are limited by throughput, large media volumes, and static conditions including an absence of hemodynamic shear. The PREDICT96 platform used here provides the advantages of throughput for other MPS in its class by incorporating 96 individual bilayer devices into a single plate, low volumes (~ 2.1 μl within each microfluidic channel itself; total recirculating volume within the channel is tunable between 60 and 150 µl), and the ability to provide flow in each channel of the device using a custom-designed “lid” containing 192 individual recirculating pumps (Fig. 1D–H). Our microvascular model in the PREDICT96 platform had long-term stability as demonstrated by high viability and good cell coverage up to at least 14 days in culture (Fig. 2).

One of the essential functions of the endothelium is to act as a semi-permeable barrier, allowing for the transport of small molecules into and out of the surrounding tissue^29^. Changes in EC barrier function can indicate drug-induced toxicity or disease states^30^ and therefore is an important assay to include in the vascular MPS toolkit. Particularly for vascular models, the macromolecular-based permeability assay provides several advantages over other methods for assessing barrier function, such as trans-endothelial/epithelial electrical resistance (TEER). Firstly, due to the inherent permeability of many vascular tissues, TEER values are often low compared to background and barrier functional changes cannot be detected^31^. In our hands, we have also found this to be true both in Transwell-based vascular models and our PREDICT96 vascular model. Therefore, although versions of the PREDICT96 platform have integrated TEER^20^, we did not include TEER as a measurement in the vascular model described here. However, by utilizing fluorescent tracer molecules of various molecular weights, we can better simulate molecular transfer in vivo. Two different molecular weights were chosen for this work: 20 kDa, corresponding to middle-sized molecules such as VEGF (21 kDa) or TNFα (25.6 kDa); and 70 kDa, corresponding to larger molecules such as albumin (66.5 kDa). We found that both dextrans were suitable for assessing our vascular model, as we could detect differences in barrier function in EC mono-cultures vs. EC-PC co-cultures as well as barrier disruption with cytochalasin B (Fig. 3). Given the standard interface of the PREDICT96 platform, the permeability assay is amenable to high throughput and robotic applications, as it merely requires pipetting samples from a 384 well plate format at the desired time points.

Imaging-based readouts are a mainstay of various biological assays, including those utilized in the pharmaceutical industry. From capturing mechanisms of action to validating a potential target to phenotypic characterization with high content screening platforms, imaging of the system is a pivotal attribute required in the drug discovery pipeline. We demonstrated the ability to screen a number of variables (10 media formulations, 2 EC sources, mono- and co-culture tissue models) in a single PREDICT96 plate in order to gain insight into their effects on the maintenance of EC monolayers. Automated image processing to capture the region of interest (i.e., the overlap area of device channels) allowed for rapid quantification of EC coverage and nuclear counts. Further expansion of these image processing capabilities, such as cellular morphology or phenotype screening, will be valuable in future work. Taken together, our results demonstrate that the PREDICT96 platform allows for high quality images which can be used for robust imaging processing and quantitative analysis to produce high content data (Fig. 4).

Modern molecular techniques provide a more comprehensive look at cellular behavior in tissue models compared to immunofluorescence-based observations, which are limited by spectral availability. High-throughput ‘omics approaches enable researchers to examine tissue cultures at every scale, including the transcriptome and the proteome. We have demonstrated the ability to isolate and measure both cellular transcripts and secreted proteins from the PREDICT96 platform. We were able to isolate RNA in sufficient quantity and quality for downstream analysis by qRT-PCR, as demonstrated here, as well as the potential next generation sequencing (NGS) applications such as RNA-seq. We have also shown the capability to measure various secreted factors, such as described in our Luminex results (Fig. 5). Most significantly, we were able to isolate RNA and secreted factors independently from each channel of the same device. For example, PDGF isoforms tended to concentrate in the EC channel, while VEGF was primarily found in the PC channel in baseline co-cultures. Previous parallel flow plate co-culture models using ECs and vascular smooth muscle cells (VSMCs) showed higher PDGF-BB levels in ECs compared to VSMCs^32^, while VEGF is an important component involved in regulation of vessel stability by PCs^33^. In regards to qRT-PCR, transcripts from the EC channel showed significant enrichment for EC markers, while the PC channel showed significant enrichment for PC-associated genes (approximately 100-fold enrichment in co-cultures vs. 1000-fold enrichment in mono-cultures) (Fig. 5B). Overall, this system provides a powerful tool for studying and isolating the impacts of co-culture interactions between distinct cell populations.

Given that the presence of multiple cell types can significantly alter tissue response, complex interactions between cell types should be considered in the development of human-based tissue models for drug screening. We applied the above molecular assays to study EC response in mono- and co-culture conditions to inflammatory insults using TNFα and low FSS. EC behavior under these perturbations in vitro has been well characterized^34^. We showed that cytokines IL-6, G-CSF, and Fractalkine were significantly increased after TNFα treatment, as demonstrated by others^35,36,37,38^. Interestingly, we found that IL-6 was significantly decreased in media isolated from ECs that had been in co-culture with PCs (Fig. 6). EC monolayers were also more robust in the co-culture conditions compared to mono-culture (Supplementary Fig. S4). Decreased cytokines and improved monolayers in the co-cultures subjected to TNFα suggests that PCs help stabilize the ECs in the presence of perturbations, as others have shown^18^. When low FSS was applied for 24 h, VE-Cadherin was significantly down-regulated, while IL-6 was significantly up-regulated in the EC channel for both mono- and co-cultures, as previously demonstrated^39^. We did not observe a change in KLF2, eNOS, or PECAM-1 gene expression, likely because these genes become responsive at higher FSS^40,41,42,43^. In the co-culture subjected to low FSS, IL-6 gene expression was significantly increased over mono-culture, indicating that the presence of PCs may enhance the inflammatory response in this case. We note that this work with the current PREDICT96 pump design is restricted to low FSS conditions; however, it is an important first step towards a high shear platform under development for future vascular model iterations. In addition, it is important to note that the co-culture devices in the current PREDICT96 plate design contain regions of mono-cultures in the device “arms” that flank the co-culture overlap region. These mono-culture regions could have different characteristics than the co-culture overlap region, which may influence cellular responses. Certain methods allow assessment isolated to the overlap region only (such as microscopy, the permeability assay, or other in situ methods), while other “bulk” readouts (such as secreted factors in the media by Luminex or gene expression by RT-qPCR) will include contributions by the mono-layer populations. In the case of the bulk analyses, the effect would be to blunt the responses we observed; nevertheless, we were still able to detect differences between our two different model configurations. Alternative device designs could maximize the co-culture overlap region to further enhance differences, which could be explored in future work.

## Conclusion

The high-throughput PREDICT96 platform, microvascular co-culture model, and assays described in this paper provide a robust system and corresponding toolkit for studying interactions between distinct cell types in bilayer microfluidic devices. The methods for macromolecular permeability and imaging-based screening are readily scalable to the platform’s throughput. In particular, the ability to study cell type-specific secreted factors and gene expression patterns are especially powerful for gaining insight into interactions between distinct cell populations and cell-specific responses. Future applications of the model and platform include the development of a high FSS pumping system and vascular injury or disease models.

## Methods

### Design and fabrication of the PREDICT96 platform and pumps

The 96 arrayed bilayer microfluidic platform was designed to be compatible with standard microplate technology. Each device of the array is comprised of two microfluidic channels (top and bottom) that are separated by a microporous polycarbonate track-etched (PCTE) membrane (Sterlitech Inc.) with a nominal pore diameter of 3 µm and pore density of 2 × 10^6^ pores/cm^2^. The inlets and outlets of the top and bottom channels each interface with one well of a 384 well-plate. The channels, which have a width of 1 mm and a height of approximately 250 µm, were fabricated using an ultrafast laser to cut laminated films of thermoplastic cyclo-olefin (co)polymer (COP/COC). The films were subsequently aligned and assembled along with the PCTE membrane and the bottomless 384 well plate, which were bonded through a thermal fusion process at 120 °C and 175 psi in a hydraulic press (Carver).

The PREDICT96 pump lid is an array of 192 individual pneumatically-driven pumps that is capable of controlling fluid flow in the top and bottom channels of devices at independent flow rates. The pumps function by recirculating the media from the inlet well to the outlet well of the channel of an individual device. This establishes a hydrostatic pressure differential that provides an equal flow rate to that of the pump in the microfluidic channel (top or bottom) connecting the two ports. Each individual pump is comprised of a fluidic and a pneumatic circuit that are separated from one another by a 25 µm-thick polyimide membrane. The fluidic circuit of the pump is made up of a pump chamber with two valves on either side of the chamber. A custom pneumatic manifold that provides vacuum and pressure actuates the polyimide membrane over the valves and the pump chamber in a peristaltic sequence through the pneumatic circuit, which in turn generates flow in the fluidic circuit of the pump. The timing of this sequence is prescribed by a custom controller that coordinates the action of a bank of 3-way solenoid valves that switch between open (vacuum) and closed (pressure) states of the valves and pump controllers. Flow parameters are set by the user via a simple graphical interface.

The pumps were fabricated using methods previously described^44^. In brief, the films comprising the fluidic and pneumatic layers (Kapton polyimide, Ultem polyetherimide, and Viton) were prepared by annealing and tacking an adhesive film (RFlex 1000 with a thickness of 12.5 µm) when necessary and through-cut using a UV laser system (LPKF). The layers were subsequently assembled and laminated at 175 °C in a heated press. To calibrate the pumps, a fluorescent solution of 6 μM fluorescein (Millipore Sigma, Burlington, MA) in PBS was pumped through the device in a 384 well-plate (Aurora Microplates) for a set number of cycles. The output was measured using a plate reader (SpectraMax) to determine the stroke volume of each of the 192 pumps. The average stroke volume and pumping frequency are used to set flow rates in the devices.

### Cell culture maintenance and co-culture media

Primary human retinal microvascular endothelial cells (ECs) were purchased from Angio-Proteomie (Boston, MA) and expanded in the manufacturer’s Endothelial Growth Medium (EGM). Primary human dermal microvascular ECs were purchased from Lonza (Basel, Switzerland) and expanded in the manufacturer’s recommended medium EGM-2MV (EBM™-2 Basal Medium and Microvascular Endothelial Growth Medium SingleQuots™ supplements). Immortalized human retinal pericytes (PCs) were generated by Pfizer and expanded in Angio-Proteomie’s Pericyte Growth Medium (PGM). All cells were used at passage 6 for the experiments described below.

For EC mono-culture and EC-PC co-culture studies in PREDICT96 platforms, ECs were seeded in MCDB131 Complete Medium composed of MCDB131 base media (ThermoFisher Scientific, Waltham, MA), microvascular growth supplement (ThermoFisher), 1 × GlutaMax (ThermoFisher), and 1 × Penicillin–Streptomycin (ThermoFisher). The ten starvation media formulations screened in co-culture contained various concentrations and combinations of the following in MCDB131 base: fetal bovine serum (Life Technologies, Carlsbad, CA), hydrocortisone (Millipore Sigma), heparin (StemCell Technologies), basic FGF (PeproTech, Inc., Rocky Hill, NJ), EGF (PeproTech), dibutyryl cAMP (R&D Systems), insulin-transferrin-selenium (ThermoFisher), L-ascorbic acid 2-phosphate (Millipore Sigma), chemically defined lipids (Millipore Sigma), knock out serum replacement (ThermoFisher), MITO + Serum Extender (Corning).

### Preparation of PREDICT96 plates for cell seeding

Prior to cell seeding, the PREDICT96 plates were treated in an oxygen plasma system (March Instruments, Inc.) for 3 min at 100 W to render the cell culture surfaces hydrophilic. The plates were then sterilized by 20 min UV exposure while dry. Following UV treatment, the plate was washed with 70% ethanol by sequentially adding 100 μl to the inlet ports and 15 μl to the outlet ports of both channels of the devices. The height differential of these volumes drives fluid flow through the channels. Each device was then washed twice with sterile distilled water, followed by sterile PBS using the same volume differentials. The microfluidic channels were then coated with Fibronectin (Millipore Sigma) at 5 µg/mL in PBS for 1–2 h at 37 °C. Immediately prior to cell seeding, the devices were primed with cell culture media.

### Seeding of EC and PC in PREDICT96 plates

ECs were harvested from T75 flasks using Accutase. After a 3–5 min incubation, cells were collected in serum-containing media and centrifuged at 220Xg for 5 min. The cell pellet was re-suspended in MCDB131 Complete Media for counting and adjusted to a final density of 1 × 10^6^ cells/mL. Immediately prior to seeding, the priming media was gently aspirated from all PREDICT96 device ports, followed by the addition of 15 μl media to the inlet and outlet ports of the bottom channel only. ECs were then seeded into the top channel only by adding 35 µl/15 μl to inlet/outlet ports. After the port volumes reached equilibration, the plate was incubated at 37 °C for 2–3 h to allow cell attachment. Afterward, the media was replaced with fresh MCDB131 Complete Media and changed every other day until PC seeding (3–5 days).

Retinal PCs were harvested using similar methods as described above and re-suspended at a density of 500,000 cells/ml. For seeding into the bottom channel, media was first aspirated from all ports, and 15 μl of fresh MCDB131 Complete Media was added to the inlet and outlet ports of the top channel. PCs were then seeded into the bottom channel by adding 35 µL/15 μL to inlet/outlet ports. Immediately after confirming flow-through and equilibration of the volumes (about 2–3 min), the plate was flipped upside down to maximize cell adhesion to the bottom surface of the membrane. The plate was then carefully transferred to an incubator for 2 h to allow PCs to adhere. Afterward, the plate was flipped right side up, and the media was refreshed in both channels of the devices.

### Live cell tracking and viability assays

Using the methods above, ECs and PCs were seeded into a PREDICT96 plate to monitor co-culture viability over a 2-week period. Devices were assessed at 24 h post-EC seeding, and then at days 7 and 14 after PC seeding. Viability was assessed using a Live-Dead assay kit (ThermoFisher). At the conclusion of the experiment on day 14, remaining samples were fixed for staining as described below.

### Immunocytochemistry and confocal imaging

Devices were fixed in cold methanol and acetic acid (95%/5% vol/vol) for 15 min at 4 °C and washed three times with PBS. Samples were then blocked with 3% normal goat serum (NGS) (ThermoFisher) for 60 min at room temperature. Primary antibody for PECAM-1 (Abcam, rabbit polyclonal) was diluted 1:250 in 3% NGS and incubated overnight at 4 °C on a rocker. The samples were washed three times with 3% NGS for 5 min each with rocking and then Alexa Fluor 488 goat anti-rabbit IgG (ThermoFisher) was added with Hoechst 33,342 nuclear stain (1 mg/ml stock, ThermoFisher) at 1:250 dilution and Alexa Fluor 647 conjugated to Phalloidin (Abcam) at 1:1000 dilution in 3% NGS. After 1 h, the samples were washed at least three times for 5 min with PBS prior to imaging.

The stained PREDICT96 devices were imaged using a Zeiss LSM700 laser scanning confocal microscope and Zen Black software. Tile scans of channel overlap areas at 10 × magnification were acquired.

### Permeability assay with FITC-dextran

The permeability assay was performed on EC mono-cultures and EC-PC co-cultures after 4 days. A solution containing 50 μg/ml FITC-dextran (20 kDa and 70 kDa) (ThermoFisher) was prepared in MCDB131 Complete Media. Cytochalasin B (Millipore Sigma) at 5 μg/ml was also prepared for barrier disruption assays. While 100 μl of FITC-dextran-containing media was applied to the top channel of each device, bottom channels were refreshed with “blank” media (no dextran). Samples of 10 μl were collected from the top and bottom channels at 0, 20, 40, 60, 120, and 260 min. In between sample collection, the cultures were incubated at 37 °C and 5% CO_2_ and placed under feeder flow to facilitate mixing (10 μl/min in both channels) using the PREDICT96 pump. Each condition had N = 8–10 replicates.

The collected samples were analyzed with a BioTek HM1 plate reader measuring at 490/520 ex/em using Gen5 software. Dextran masses were determined using a standard curve generated for each molecular weight. To determine the permeability coefficient, the following equation was used:

$$ P = \frac{{\left[ {C\left( t \right) - C\left( {t_{0} } \right)} \right] \cdot V}}{{A \cdot t \cdot C_{0} }} $$

where *C(t)* is the FITC-dextran concentration at 260 min, *C(t*_*0*_*)* is the FITC-dextran concentration at 0 min, *V* is the volume (cm^3^) of the basolateral chamber, *A* is the surface area (cm^2^) of the membrane, *t* is duration of the assay (sec), and *C*_*0*_ is the initial concentration of FITC-dextran applied to the top channel.

### Image processing and quantification

Tiled images of the devices were converted into TIF format and processed using custom Python code to output channel coverage and nuclear counts. Outputs included visual representations as well as chart formats. First, the channel edges were cleaned using decisions based on the brightness of the green and blue channels as well as the differences between brightness of the channels. Next, the image was run through connected-component-analysis which breaks the image into connected sections. This identified the channel overlap area as one of the connected components. Next, a series of decisions discarded the non-overlap sections, leaving the channel overlap area as a binary image or mask. The binary image of the channel overlap was then combined with the original image to mask out everything but the region of interest: the cells within the channel overlap.

Once the image was masked, the PECAM-1 and Hoechst channels were analyzed separately to ascertain the percent cell coverage. For both mono-cultures and co-cultures, the green channel was thresholded and used for identification of the area covered by ECs. The threshold was calculated differently for mono- vs. co-cultures. In the case of the mono-cultures, the Hoechst channel was used to identify the nuclei. The nuclear objects were dilated to bridge the gaps between the nuclei and the PECAM-1 border stain of the ECs. After the thresholding was completed, the number of identified pixels was divided by the total number of pixels in the channel, calculating the percent coverage of ECs in the channel overlap. The Hoechst channel was also thresholded and segmented using connected component analysis, and processed to count the nuclei (accounting for clusters of nuclei).

The cell coverage image was overlaid onto the original PECAM-1 image for a combined visual output. A similar overlay was made from the original Hoechst stain overlaid with the identification of nuclei. Calculations of EC coverage and nuclei were also generated as Excel files that matched the format of the PREDICT96 plate.

### Inflammatory assay and channel-specific cytokine profiles

EC mono-culture, PC mono-culture, and EC-PC co-culture conditions were established in a PREDICT96 plate as described above. At 24 h post-PC seed, the samples were switched to Starvation Medium. After another 24 h, samples were subjected to TNFα stimulation as follows. Acute stimulation conditions were treated with 10 ng/mL TNFα (R&D Systems) for 4 h, followed by fresh Starvation Medium without TNFα for a 20 h recovery period. Chronic stimulation conditions were treated with a single dose of 10 ng/mL TNFα and incubated for 24 h. Controls included samples with and without the addition of vehicle (0.1% BSA in PBS). Media was collected from the top and bottom channels of the devices and immediately frozen at − 80 °C until use. Immediately after media collection, devices were fixed and stained as described above to evaluate EC monolayers.

A custom Magnetic Luminex Performance Assay kit containing a pre-mix of 12 analytes of interest (R&D Systems, Human XL Cytokine Discovery Panel) was used: CCL2/MCP-1, fractalkine/CX3CL1, G-CSF, GM-CSF, IL-6, IL-8/CXCL8, IL-10, IL-13, IL-33, PDGF-AA, PDGF-BB, and VEGF. Undiluted media samples collected from devices were run according to the manufacturer’s protocol and processed using a Luminex FLEXMAP 3D and xPONENT software (version 4.2). The multiplex data were analyzed using Milliplex Analyst (version 5.1, Vigene Tech Inc.) to generate standard curves using a five parameter logistic (5-PL) curve-fit to determine the concentration of each analyte in the sample. To determine the effects of TNFα stimulation, treated samples were normalized to untreated controls and presented as a fold-change.

### Application of fluid shear stress in PREDICT96 devices

EC mono-culture, PC mono-culture, and EC-PC co-culture conditions were established in PREDICT96 plates as described above. Following EC seeding, feeder flow at 0.01 dyn/cm^2^ was applied to the top channel of devices using the PREDICT96 pump, and transiently paused for PC introduction into devices. After a 24 h starvation period, low FSS at 0.5 dyn/cm^2^ was applied to the top channel of devices. Note that the bottom channel containing PCs remained static for the duration of the experiment. Static controls were run on a separate PREDICT96 plate. After 24 h, RNA was extracted from device channels as described below. Samples were then immediately fixed and stained to assess EC monolayers.

To estimate the flow rates needed for the target FSS above, laminar flow in a rectangular channel was assumed. Feeder flow at 0.01 dyn/cm^2^ and low FSS at 0.5 dyn/cm^2^ equated to flow rates of 1 μl/min and 48 μl/min, respectively.

### RNA extraction from devices and channel-specific qPCR

For qPCR studies, devices were washed three times with PBS to ensure removal of serum. Accutase was added to devices in both the top and bottom channels and incubated at 37 °C for 3 min. After the Accutase was recovered, each channel of the devices was flushed three times with fresh media to remove the remaining cells. These media washes were pooled with the recovered Accutase after each flush. The pooled Accutase/media containing the harvested cells was spun down for 5 min at 2000×*g* to obtain the cell pellet. The supernatant was discarded and RNA was subsequently extracted using the Qiagen RNeasy Micro Kit according to the manufacturer’s specifications. RNA quality was evaluated using the Agilent TapeStation with High Sensitivity RNA screentapes.

Complementary DNA (cDNA) was synthesized using the SuperScript IV VILO Master Mix with ezDNAse Enzyme (Thermo Scientific) according to the manufacturer’s specifications. Quantitative PCR was performed using Taqman reagents (Thermo Scientific). Briefly, 1 μl of cDNA was mixed with 0.5 μl of the appropriate Taqman probe (Table 1) and 5 μl of Taqman Fast Advanced Master Mix (Thermo Scientific) and water for a final volume of 10 μl per well in a 384 well plate. The reaction was run in an Applied Biosystems QuantStudio 7 Flex System (Thermo Scientific) using the following cycle: 20 s at 95 °C followed by 40 cycles of 95 °C for 1 s then 60 °C for 20 s. Comparative C_T_ values were determined using the method described by Schmittgen and Livak^45^ using GAPDH as a reference gene.

**Table 1:** List of genes and probes used.

Transcript	Taqman probe	Cell type	Function
KLF2	Hs00360439_g1	EC	Transcription factor responsive to fluid shear stress/pro-inflammatory stimuli
NOS3 (eNOS)	Hs01574665_m1	EC	Production of nitric oxide
PECAM1	Hs01065279_m1	EC	Intercellular adhesion molecule
CDH5 (VE-Cadherin)	Hs00901465_m1	EC	Intercellular adhesion molecule
IL-6	Hs00174131_m1	EC/PC	Pro-inflammatory cytokine
CSPG4 (NG2)	Hs00171790_m1	PC	Angiogenesis
PDGFRβ	Hs01019589_m1	PC	Recruitment

*EC* endothelial cell; *PC* pericyte.

### Statistical analysis

Data are presented as mean ± standard deviation and were analyzed using GraphPad Prism version 7.04 for Windows (GraphPad Software, La Jolla California, USA, www.graphpad.com). Statistically significant outliers were determined using the Grubb’s test. Statistical significance was determined using the Student t-test for pairwise comparisons, and two-way or three-way analysis of variance (ANOVA) with Tukey’s post hoc test for multiple comparisons, where appropriate. A p-value lower than 0.05 was considered statistically significant and is indicated in figures as follows: * P ≤ 0.05, ** P ≤ 0.01, *** P ≤ 0.001, and **** P ≤ 0.0001.

## Supplementary Information

Supplementary Information.

## Data Availability

The datasets generated during and/or analyzed during the current study are available from the corresponding author upon reasonable request.
