# Supplementary material for: A high-throughput microfluidic bilayer co-culture platform to study endothelial-pericyte interactions
Source: Sci Rep. 2021 Jun 9;11:12225. doi: 10.1038/s41598-021-90833-z (PMC8190127; doi:10.1038/s41598-021-90833-z)
Supplement: Supplementary file 1 — Supplementary Information. [file 41598_2021_90833_MOESM1_ESM.pdf]

A high-throughput microfluidic bilayer co-culture platform to study endothelial-pericyte interactions

Miles T. Rogers[1#a], Ashley L. Gard[1#], Robert Gaibler[1#], Thomas J. Mulhern[1#], Rivka Strelnikov[1#b], Hesham Azizgolshani[1], Brian P. Cain[1], Brett C. Isenberg[1], Nerses J. Haroutunian[1], Nicole E. Raustad[1c], Philip M. Keegan[1d], Matthew P. Lech[2], Lindsay Tomlinson[2], Jeffrey T. Borenstein[1], Joseph Charest[1*], Corin Williams[1*]

[#] *equal contributors/co-first authors*

SUPPLEMENTARY INFORMATION

SUPPLEMENTARY FIGURES

		20 kDa						70 kDa					
EC/RPC Co-Culture	(CB -)	2.21E-05	9.7E-06	8.31E-06	3.31E-06	1.42E-06	3.62E-06	7.26E-06	1.51E-06	3.61E-06	2.9E-06	1.65E-06	2.43E-05
		2.28E-05	2.3E-06	1.48E-06	7.52E-06	1E-06	2.81E-07	2.06E-07	4.2E-07	1.19E-07	1.43E-06	1.43E-06	2.42E-05
	(CB +)	2.35E-05	1.8E-05	1.4E-05	1.68E-05	1.1E-05	9.76E-06	7.8E-06	5.21E-06	8.6E-06	8.84E-06	1.32E-05	2.26E-05
		2.32E-05	1.3E-05	1.14E-05	1.2E-05	1.02E-05	1.11E-05	7.31E-06	5.58E-06	8.93E-06	4.53E-06	1.05E-05	2.29E-05
EC Mono-Culture	(CB -)	7.32E-07	5.8E-06	1.64E-05	9.17E-06	1.58E-05	7.89E-06	6.3E-06	1.46E-05	3.1E-06	3.1E-06	3.41E-06	3.71E-05
		3.8E-05	2.5E-05	3.5E-06	3.16E-06	3.69E-06	1.07E-05	1.08E-05	4.81E-06	6.03E-06	9.71E-06	6.19E-06	3.56E-05
	(CB +)	3.31E-05	2.7E-05	2.61E-05	2.16E-05	1.69E-05	1.88E-05	2.29E-05	2.43E-05	2.06E-05	2.53E-05	2.49E-05	3.6E-05
		4.05E-05	2.4E-05	1.98E-05	2.11E-05	2.46E-05	2.54E-05	2.95E-05	1.71E-05	1.17E-05	2.35E-05	2.21E-05	3.94E-05
		blank											*blank*

Supplementary Figure 1: Permeability coefficient heat map. Heat map of permeability coefficients resulting from permeability assay run on PREDICT96 plate. Conditions include blank devices (no cells), endothelial cell mono-culture and endothelial-pericyte co-culture, with or without cytochalasin B (CB).

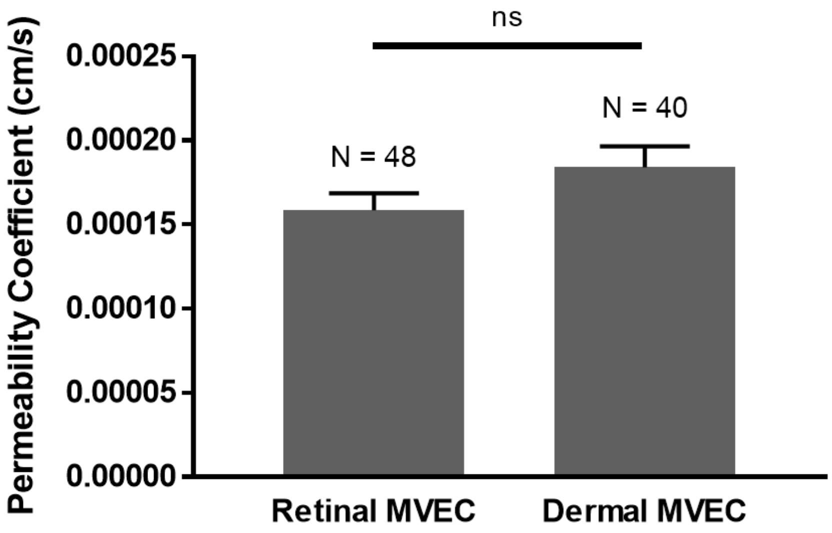

Supplementary Figure 2: Permeability coefficients for media screening study. Permeability coefficients were calculated for each microvascular EC type in mono-culture (20 kDa FITC-dextran, 60 min), prior to seeding of retinal PCs for media screening. There was no significant difference in barrier integrity between the two EC sources ($p = 0.12$).

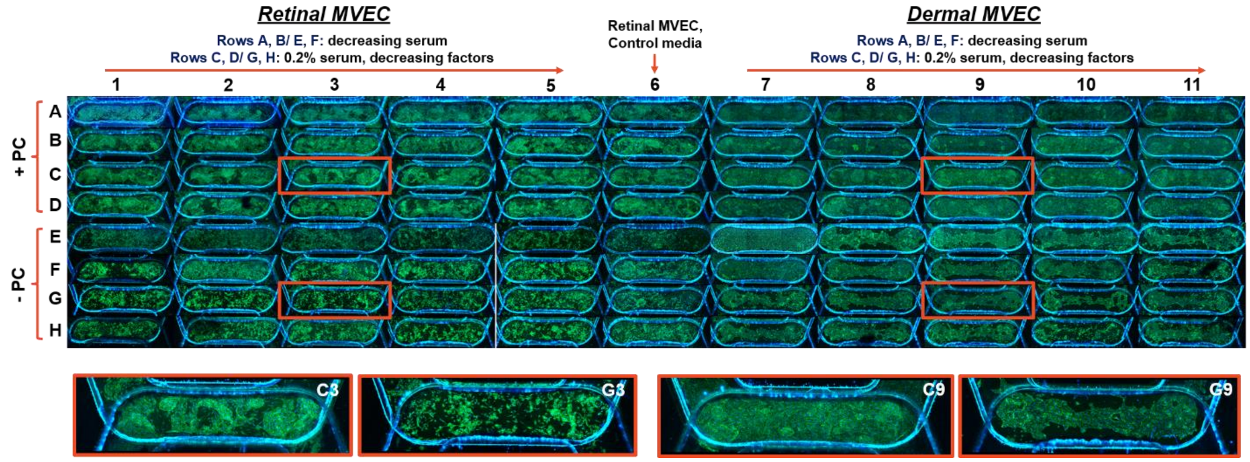

Supplementary Figure 3. Screening of EC source and media in mono- and co-culture. Images shown for a screening study of EC monolayer health under various conditions. Note column 12 was left blank for permeability assay (images not shown). Select images are highlighted to show the difference in EC monolayer in co-culture vs. mono-culture. These images were used for quantification outputs shown in Figure 4.

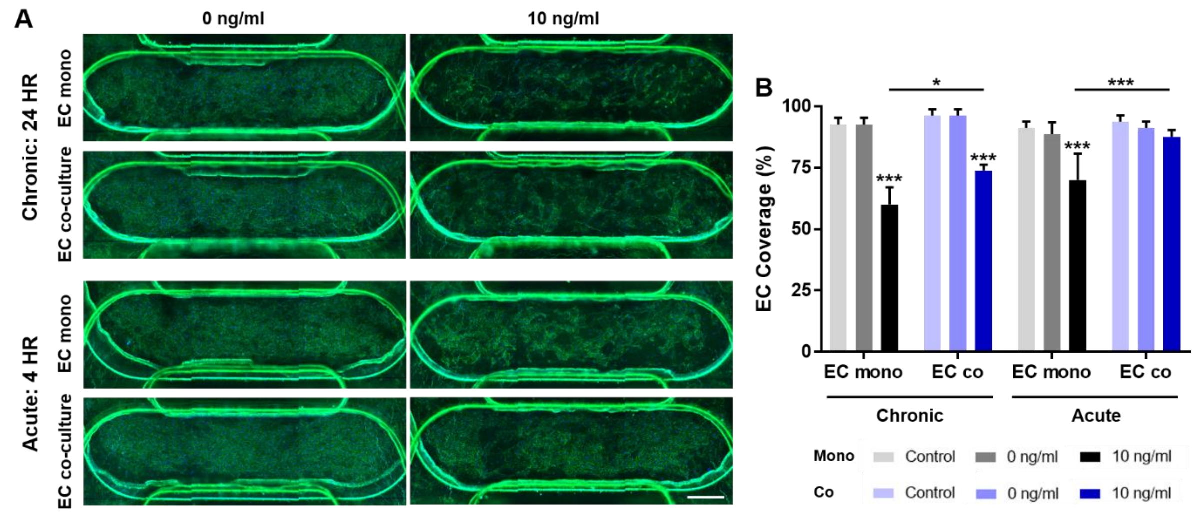

Supplementary Figure 4: Effect of TNFα stimulation on EC monolayers. (A) Representative images of device overlap area at 24 hr post-stimulation. (B) Quantification of EC coverage. N = 4 devices per condition analyzed. Scale bar = 500 µm.
